# Enzymatic origin and various curvatures of metabolic scaling in microbes

**DOI:** 10.1038/s41598-019-40712-5

**Published:** 2019-03-11

**Authors:** Liyan Li, Genxuan Wang

**Affiliations:** College of Life Sciences, Zhejiang University, Hangzhou, China

## Abstract

The famous and controversial power law is a basal metabolic scaling model mainly derived from the “surface rule” or a fractal transport network. However, this law neglects biological mechanisms in the important active state. Here, we hypothesized that the relative metabolic rate and growth rate of actively growing microbes are driven by the changeable rate of their rate-limiting enzymes and concluded that natural logarithmic microbial metabolism (ln*λ*) and growth (or biomass) (ln*M*) are both dependent on limiting resources, and then developed novel models with interdependence between ln*λ* and ln*M*. We tested the models using the data obtained from the literature. We explain how and why the scaling is usually curved with the difference between microbial metabolic and growth (or biomass’s) half-saturation constants (*K*_*M*_, *K*_λ_) in the active state and agree that the linear relationship of the power law is a particular case under the given condition: *K*_*M*_ = *K*_λ_, which means that the enzyme dynamics may drive active and basal metabolic scaling relationships. Our interdependent model is more general than the power law, which is important for integrating the ecology and biochemical processes.

## Introduction

Since Louis Pasteur first performed quantitative studies of microbial growth at the dawn of microbiology in 1857^1,2^, microbes have been subjected to more complete and accurate studies on their growth kinetics, physiology and metabolism^3–5^ over a century. Within this historical context, microbial growth dynamics models, which simulate the behaviors of microbial growth dynamics and the development of system architecture^6^, have been applied to the various large-scale processes^7–9^. Among these models, the farthest-reaching coarse-grained model is Monod’s equation, an unstructured model, describing functional relationships between the specific growth rate (*μ*) in a culture and a single essential growth-limiting substrate concentration (C_s_). The model may take the following forms^10^:

1 $$\frac{1}{{C}_{x}}\frac{d{C}_{x}}{dt}=\mu =\frac{{\mu }_{\max }\cdot {C}_{s}}{{K}_{s}+{C}_{s}}$$

or

2 $$\frac{1}{M}\frac{dM}{dt}=\mu =\frac{{\mu }_{\max }\cdot {C}_{s}}{{K}_{s}+{C}_{s}}$$

in which *C*_*x*_ is the concentration of microbial cells, *M* is the biomass of microbial cells, d*C*_*x*_/d*t* and dM/d*t* are the microbial growth rate, *μ*_max_ is the maximum specific growth rate, and *K*_s_ is the Monod constant, i. e., the half-saturation constant for the substrate.

Many people regarded the Monod equation as a theoretical one describing the relationship between the microbial specific growth rate (or biomass) and limiting resources, as the Monod equation is in the same form as the Michaelis-Menten equation, which is one of the most famous models of enzyme kinetics and includes constants with mechanistic meaning^11,12^. However, the Michaelis-Menten equation was derived from the mechanism of enzymatic reaction, while the Monod equation was developed from a curve-fitting exercise. The Monod equation is purely empirical and lacks a theoretical basis^13^, so none of the Michaelis-Menten constants, which are appropriate for an enzyme-substrate system, can be applied to a substrate-cell system^14^. Over the last few decades, numerous researchers have devoted great efforts to proposing many powerful theoretical interpretations to support the Monod equation, such as the thermodynamics of a microbial growth process^13^, and mass transfer concentrations^14^. A new mechanical model of the relationship between microbial growth and limiting resources may be helpful for deepening the understanding of the microbial growth kinetics.

Metabolism, a collection of chemical transformations for maintaining life in a living cell, includes the biological processes that enable the exchange of material and energy between the body and the outside world and the self-renewal process of matter and energy in the body^3^. A long time ago, researchers discovered a certain relationship between metabolic rate and body size (or biomass), in which small-bodied organisms generally have higher mass-specific metabolic rates than larger-bodied organisms. The same is true at the unicellular level for free-living single-celled microbes^15,16^. Much progress has been made in understanding metabolism via allometric studies of many small organisms^17–19^. Furthermore, the relationship between body size and metabolism known as the power law^20^, is one of the most fundamental features of life, with scaling as presented in Eq. (3):

3 $$\lambda ={\lambda }_{0}{M}^{a}$$

where *λ* is the whole-organism basal metabolic rate (in watts or another unit of power), *λ*_0_ is a normalization constant that is independent of body size or temperature, *M* is organismal volume (often expressed in biomass, in kg), and *α* is a scaling exponent.

In 1932, based on animal data, Kleiber concluded that the exponent *α* is a constant equal to 3/4^20^. Then, to explain why *α* is equal to 3/4, the metabolic theory of ecology (MTE) was put forward by Brown, West, Enquist and colleagues^21–23^. The power law is predominant in much empirical literature, but the MTE is not applicable to microbes^5,16^, mainly because of the value of *α*^24–26^, which increasing lines of evidence suggest that α is not equal to 3/4, for example, in some small unicellular organisms, the value of *α* is greater than 3/4^19,27^. The scaling exponent varies not only between taxa but also between cells of an individual species and between species of the same taxonomic group^28,29^. It is clear that α is variable and inconsistent with an assumption underlying the MTE^28^, and *λ*_0_ also exhibit a very large range of variation^30,31^. Both *λ*_0_ and *α*, which shift dynamically in plants, animals and microbes, are convincingly influenced by limiting resources, including water, food, and oxygen etc^32–34^, as are metabolism and body mass. Many researchers have used the concept of the Michaelis-Menten equation^12^ to describe the relationship between metabolism and limiting resources^35–37^, which like the form of the Monod equation, lacks theoretical support and an underlying biological mechanism. Is there a mechanical and theoretical model for the relationship between microbial metabolism and limiting resources?

Scientists have long sought to establishing a universal quantitative theory explaining the correlation between metabolism and growth in organisms. Since the allometric growth relationship was put forward^38^, increasing numbers of researchers have become interested in this pursuit. In microbiology, microbial growth and metabolism have been extensively and separately investigated^3–5^. Generally, researchers investigated the relations of microbial growth (or biomass) to metabolic rate usually using the power equation (Eq. 1), so a linear relationship between metabolic rate and biomass in microbes has been detected in many studies^16,39^. Nevertheless, there has been disagreement about the cause *versus* effect relationship between these variables over time, leading to the conclusion of four not necessarily mutually exclusive possibilities: metabolism drives growth; growth drives metabolism; growth and metabolism affect each other by reciprocal feedback; and (or) growth and metabolism are similarly, but independently related to a third factor or set of factors^40^. Every possibility has its own supporters who provide many experimental and theoretical lines of evidence in plants, animals and microbes to support their hypotheses^41–45^, so it is difficult to disentangle cause vs. effect. Therefore, the questions of whether there is a certain relationship between microbial metabolism and growth and which is the dominant force between them both remain unanswered. Given these unanswered questions and the variation in *λ*_0_ and *α*, will the relationship between microbial metabolism and growth still be linear or follow the power equation?

In fact, the traditional power law is a model that is specifically used for the basal (or inactive) metabolic rate; thus, the log-log relationship between metabolic rate and body size (or biomass) is linear. If the metabolic rate is active, what is the result? Previously, Delong *et al*.^29^ showed that active and inactive metabolic rates scale linearly with body mass based on data collected at the interspecies level. Therefore, to obtain a more general understanding of microbial growth and metabolism, we aimed to synthesize data from published studies. In the present study, at the interspecific level, we developed resource-dependent equations of natural logarithmic microbial metabolism (ln*λ*) and growth (or biomass) (ln*M*) and then obtained equations interdependent between ln*λ* and ln*M* based on the hypothesis that the relative metabolic rate and the relative growth rate in microbes are driven by their own rate-limiting enzymes, which is generally supported by data compiled from many articles on microbes. We found that active metabolic scaling is generally nonlinear, its curvature is derived from the difference between microbial metabolic and growth (or biomass’s) half-saturation constants (*K*_*M*_, *K*_λ_) in the active state, and particularly, for the same values of *K*_*M*_ and *K*_λ_, there is a linear scaling relationship. Therefore, we argue that a power law based on the basal metabolism may be the particular dynamics in our new interdependent model, which means that, at least in microbes, enzyme dynamics rather than the surface rule or a fractal resource transport network^21,46,47^ is a main driver of the active and basal metabolic scaling curvatures.

## The Enzyme-Driven Metabolic Scaling Model

### The resource-dependent active metabolism model

Metabolism is a collection of chemical transformations and enzyme-catalyzed reactions that perform a variety of functions, ranging from nutrient breakdown to the polymerization of macromolecules. It is reasonable to hypothesize that the relative metabolic rate of microbes (*dλ/λ*) is constrained by the key enzymatic rate of their metabolism^48^, under the conditions that other factors are constant over time:

4 $$\frac{d\lambda }{\lambda }=d{v}_{\lambda }$$

where *λ* is the active metabolic rate, *v*_*λ*_ is the key enzymatic rate of the metabolism, *dλ* is the differential of *λ* and *dv*_*λ*_ is the differential of *v*_*λ*_.

Equation (5) on the relationship between the metabolic rate and the key enzymatic rate of metabolism was obtained by integrating Eq. (4):

5 $$\lambda =a{e}^{b{v}_{\lambda }}$$

where *a* is the coefficient of transformation, and *b* is the efficiency of *v*_*λ*_.

Eq. (6) on the relationship between the metabolic rate and the concentration of a limiting resource was obtained by taking the logarithm and substituting the Michaelis-Menten equation^12^ into Eq. (5):

6 $$\mathrm{ln}\,\lambda =\frac{\mathrm{ln}\,{\lambda }_{\max }\cdot {C}_{s\lambda }}{{K}_{\lambda }+{C}_{s\lambda }}+\,\mathrm{ln}\,a$$

where *C*_sλ_ is the concentration of a limiting substrate, ln *λ*_*max*_ = b*V*_λ_ is the maximum metabolic rate when *C*_sλ_ approaches saturation and other resources remain constant, *V*_λ_ is the maximum rate of the key enzymatic reaction of the metabolism in the Michaelis-Menten equation, and *K*_λ_ is the half-saturation constant.

### The resource-dependent growth model

Most of the reactions that occur during microbial growth are enzymatic reactions. It is reasonable to hypothesize that the relative rate of microbial biomass (the specific growth rate) is constrained by the key enzymatic rate of microbial growth in each growth stage under the conditions that other factors are constant over time:

7 $$\mu =\frac{dM}{M}=d{v}_{\mu }$$

where *μ* is the specific growth rate, *v*_*μ*_ is the key enzymatic rate in the growth process, *M* is the biomass, *dM* /*M* is the relative rate of change in microbial biomass, and *dv*_*μ*_ is the partial differential of *v*_*μ*_.

Equation (8) on the relationship between the specific growth rate and the key enzymatic rate in the growth process was obtained by integrating Eq. (7):

8A $$\mu ={h}_{1}{v}_{\mu }$$

8B $$M=c{e}^{{h}_{2}{v}_{\mu }}$$

where *c* is the coefficient of transformation, and *h*_1_, *h*_2_ is the efficiency of the key enzymatic rate in the growth process.

Equation (9) on the relationship between the specific growth rate and the concentration of a growth-limiting resource was obtained by taking the logarithm and substituting the Michaelis-Menten equation^12^ into Eqs (8A) and (8B):

9A $$\mu =\frac{{\mu }_{\max }\cdot {C}_{s\mu }}{{K}_{\mu }+{C}_{s\mu }}$$

9B $$\mathrm{ln}\,M=\frac{\mathrm{ln}\,{M}_{\max }\cdot {C}_{sM}}{{K}_{M}+{C}_{sM}}+\,\mathrm{ln}\,c$$

where *C*_s*μ*,_ *C*_s*M*_ is the concentration of a growth-limiting substrate, *μ*_*max*_ = *h*_1_*V*_*μ*_ is the maximum rate in the growth process when C_s*μ*_ approaches saturation and other resources remain constant, ln *M*_*max*_ = *h*_2_*V*_*M*_ is the maximum biomass of microbial cells in the growth process when *C*_s*M*_ approaches saturation and other resources remain constant, *V*_*μ*,_ *V*_*M*_ is the maximum rate of the key enzymatic reaction of growth in the Michaelis-Menten equation, and *K*_*μ*_, *K*_*M*_ is the half-saturation constant.

### The natural logarithmic model with interdependence between microbial growth and metabolism

Eqs (10A), (10B), (11A) and (11B) were obtained by substituting Eqs (9A) and (9B) into Eq. (6) or *vice versa* under the conditions of the same types of limiting substrates for both microbial metabolism and growth and *K*_*λ*_ ≠ *K*_*M*_.

10A $$\mathrm{ln}\,\lambda =\frac{\mathrm{ln}\,{\lambda }_{\max M}\cdot (\mathrm{ln}\,M-\,\mathrm{ln}\,{c}_{1})}{{K}_{\lambda M}+(\mathrm{ln}\,M-\,\mathrm{ln}\,{c}_{1})}+\,\mathrm{ln}\,{a}_{1}$$

10B $$\mathrm{ln}\,M=\frac{\mathrm{ln}\,{M}_{\max \lambda }\cdot (\mathrm{ln}\,\lambda -\,\mathrm{ln}\,{a}_{2})}{{K}_{M\lambda }+(\mathrm{ln}\,\lambda -\,\mathrm{ln}\,{a}_{2})}+\,\mathrm{ln}\,{c}_{2}$$

where

$$\mathrm{ln}\,{\lambda }_{\max M}=\frac{{K}_{M}\,\mathrm{ln}\,{\lambda }_{\max }}{{K}_{M}-{K}_{\lambda }},\,{K}_{\lambda M}=\frac{{K}_{\lambda }\,\mathrm{ln}\,{M}_{\max }}{{K}_{M}-{K}_{\lambda }},$$

$$\mathrm{ln}\,{M}_{\max \lambda }=\frac{{K}_{\lambda }\,\mathrm{ln}\,{M}_{\max }}{{K}_{\lambda }-{K}_{M}},\,{K}_{M\lambda }=\frac{{K}_{M}\,\mathrm{ln}\,{\lambda }_{\max }}{{K}_{\lambda }-{K}_{M}}.$$

11A $$\mathrm{ln}\,\lambda =\frac{\mathrm{ln}\,{\lambda }_{\max \mu }\cdot \mu }{{K}_{\lambda \mu }+\mu }+\,\mathrm{ln}\,a$$

11B $$\mu =\frac{{\mu }_{\max \lambda }\cdot (\mathrm{ln}\,\lambda -\,\mathrm{ln}\,a)}{{K}_{\mu \lambda }+(\mathrm{ln}\,\lambda -\,\mathrm{ln}\,a)}$$

where

$$\mathrm{ln}\,{\lambda }_{\max \mu }=\frac{{K}_{\mu }\,\mathrm{ln}\,{\lambda }_{\max }}{{K}_{\mu }-{K}_{\lambda }},\,{K}_{\lambda \mu }=\frac{{K}_{\lambda }{\mu }_{\max }}{{K}_{\mu }-{K}_{\lambda }},$$

$${\mu }_{\max \lambda }=\frac{{K}_{\lambda }{\mu }_{\max }}{{K}_{\lambda }-{K}_{\mu }},\,{K}_{\mu \lambda }=\frac{{K}_{\mu }\,\mathrm{ln}\,{\lambda }_{\max }}{{K}_{\lambda }-{K}_{\mu }}.$$

Eqs (12A) and (12B) were obtained when *K*_*λ*_ = *K*_*M*_, and describe the relationship between metabolism and biomass, i.e., the specific growth rate in microbes.

12A $$\mathrm{ln}\,\lambda =d\,\mathrm{ln}\,M+\,\mathrm{ln}\,e$$

12B $$\mathrm{ln}\,M=f\,\mathrm{ln}\,\lambda +\,\mathrm{ln}\,g$$

where

$$d=\frac{\mathrm{ln}\,{\lambda }_{\max }}{\mathrm{ln}\,{M}_{\max }},\,\mathrm{ln}\,e=\,\mathrm{ln}\,a-\frac{\mathrm{ln}\,{\lambda }_{\max }\,\mathrm{ln}\,c}{\mathrm{ln}\,{M}_{\max }}.$$

$$f=\frac{\mathrm{ln}\,{M}_{\max }}{\mathrm{ln}\,{\lambda }_{\max }},\,\mathrm{ln}\,g=\,\mathrm{ln}\,c-\frac{\mathrm{ln}\,{M}_{\max }\,\mathrm{ln}\,a}{\mathrm{ln}\,{\lambda }_{\max }}.$$

Eq. (12) is obtained by taking the logarithm of equation (13):

13A $$\lambda =e{M}^{d}$$

13B $$M=g{\lambda }^{f}$$

The form of Equation (13) is similar to that of Eq. (3) and, expressly, the power law is a particular form of the interdependent law for both key enzymatic dynamics with the same half-saturation constant.

## Result

### The enzyme-driven relationship between a limiting resource and active metabolism

**Figure 1:**
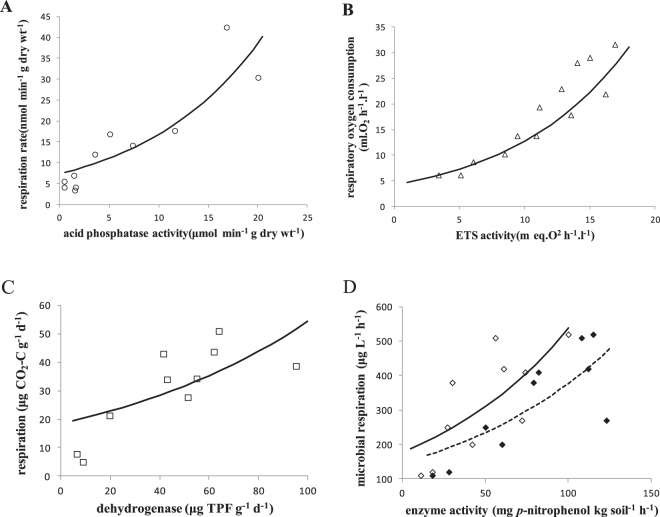
Metabolic rate increases exponentially with enzymatic activity in microbes, following Eq. (5). (**A**) Acid phosphatase activity *vs* soil respiration rate (circle) in a soil bacterium (data from Anderson *et al*.^49^). (**B**) ETS activity *vs* respiratory oxygen consumption (triangle) in the marine bacterium *Pseudomonas perfectomarinus* (data from Packard *et al*.^50^). (**C**) Dehydrogenase activity *vs* microbial respiration (square) in two Gray Luvisolic soil zones (data from Vvsr *et al*.^51^). (**D**) Enzyme activity *vs* soil bacterial respiration (hollow diamond: alkaline phosphatase, solid diamond: phosphodiesterase) (data from Frankenberger and Dick^48^). All parameter values are given in *SI Appendix* Table S2.

The basic hypothesis that the relative rate of microbial metabolism is constrained by the key enzymatic rate of the metabolism and the predictions (Eq. 5) were supported by the data compiled from several publications. The relationship between enzyme activity and metabolic rate is exponential (Fig. 1). In a soil bacterium, the soil respiration rate increased exponentially with acid phosphatase activity (Fig. 1A); the acid phosphatase activity was measured on day 7, with glucose amendment producing the highest bacterial concentrations (data from Anderson *et al*.^49^). The respiratory electron transport system (ETS) activity, which reflects the sum of the activities of nicotinamide adenine dinucleotide (NADH) oxidoreductase and succinate dehydrogenase increased exponentially with the respiratory oxygen consumption in anaerobic (tryptone-yeast extract-sea salt medium containing nitrogen, TYSN) cultures of a marine bacterium (*Pseudomonas perfectomarinus*) (Fig. 1B) (data from Packard *et al*.^50^). The dehydrogenase activity in two Gray Luvisolic soils had a positive effect on microbial respiration (Fig. 1C); the respiratory activity was determined by incubating the soil samples with a CO_2_ trap for 10 days (data from Vvsr *et al*.^51^). The soil bacterial respiration increased with alkaline phosphatase activity and phosphodiesterase activity (Fig. 1D) (data from Frankenberger and Dick^48^).

**Figure 2:**
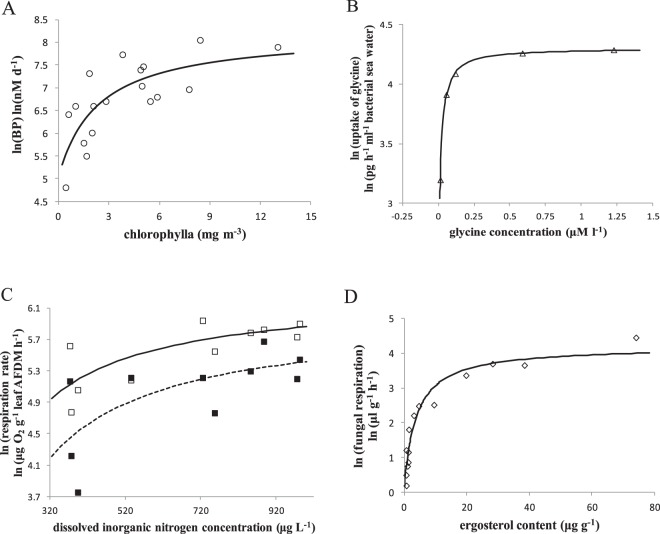
Metabolic rate increases with the concentration of limiting resources in microbes, following Eq. (6). (**A**) Bacterial production (BP) *vs* chlorophyll a in eastern waters (data from Yuan *et al*.^52^). (**B**) Substrate concentration *vs* the rate of uptake of ^14^C-glycine by marine bacteria (data from Manahan and Richardson^53^). (**C**) Dissolved inorganic nitrogen concentration *vs* microbial respiration rates associated with decaying rhododendron (hollow square) and wood veneer (solid square) leaf litter (data from Burns^54^). (**D**) Soil ergosterol content *vs* fungal respiration (diamond) in a vegetation zone (data from Imberger and Chiu^55^). The parameter values are given in *SI Appendix* Table S2.

The resources dependence of metabolic rate (Eq. 6) was tested by data on limiting resources and metabolic rate from several publications (Fig. 2). Natural logarithmic bacterial production increased with chlorophyll a in eastern waters of Hong Kong (Fig. 2A) (data from Yuan *et al*.^52^). In sea water, the trajectory of the natural logarithmic bacteria’s uptake by bacteria of ^14^C-glycine with different substrate (glycine) concentrations is curved (Fig. 2B); bacterial samples were incubated for 1 h (data from Manahan and Richardson^53^). Rhododendron leaves and wood veneers were sampled on days 28, 44, 77, 111, and 144 (data from Burns^54^). The changes in natural logarithmic microbial respiration rates formed a curve and were associated with decaying plant litter (rhododendron and wood veneer) with increasing dissolved inorganic nitrogen concentrations (Fig. 2C). Natural logarithmic fungal respiration increased with the ergosterol content (Fig. 2D) in soil samples from vegetation zones (data from Imberger and Chiu^55^).

### The enzyme-driven relationship between limiting resources and specific growth rate

**Figure 3:**
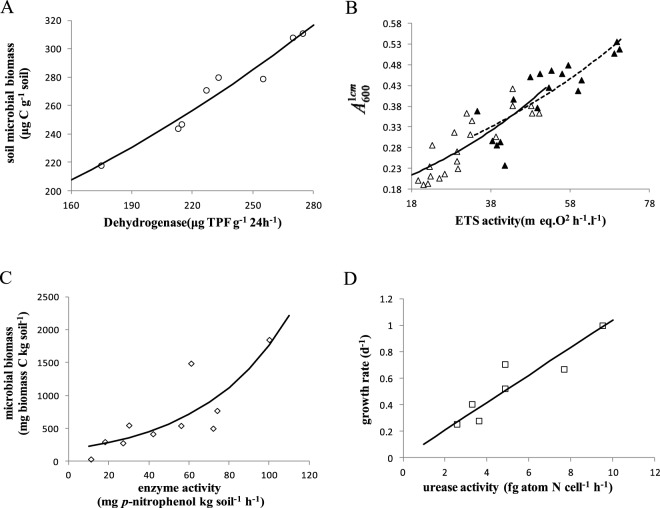
Biomass (growth rate) increases exponentially with enzymatic activity in microbes, following Eqs (8A) and (8B). (**A**) Dehydrogenase activities *vs* soil microbial biomass (hollow circle) (data from Dar^56^). (**B**) ETS activity *vs* marine bacterial biomass (hollow triangle: TYSN-${{\rm{NO}}}_{3}^{-}$, solid triangle: TYSN-${{\rm{NO}}}_{2}^{-}$) (data from Packard *et al*.^50^). (**C**) Alkaline phosphatase activity *vs* soil microbial biomass (hollow diamond) (data from Frankenberger and Dick^48^). (**D**) Urease activity *vs* the relative growth rate of *Phaeodactylum tricornutum* (hollow square) (data from Fan *et al*.^57^). The parameter values are given in *SI Appendix* Table S3.

The microbial mass-specific growth rate is the relative growth rate of microbial biomass. Equation (8) shows that the microbial specific growth rate or biomass increases linearly or exponentially with enzyme activity respectively (Fig. 3). Figure 3A shows that microbial biomass increased exponentially with dehydrogenase activity in surface samples (0–15 cm) of loam (organic C, 0.72% ; pH, 7.7) soil (data from Dar^56^). ETS activity increased exponentially with bacterial biomass in anaerobic (TYSN) cultures of a marine bacterium (*P*. *perfectomarinus*) (Fig. 3B). Biomass was monitored by measuring absorbance (A_1cm 600_) at 600 nm in a 1 cm cell. TYSN-${{\rm{NO}}}_{3}^{-}$ and TYSN-${{\rm{NO}}}_{2}^{-}$ represented two phases of respiration in anaerobic cultures of *P*. *perfectomarinus* (data from Packard *et al*.^50^). The relationship between alkaline phosphatase activity and soil microbial biomass is exponential (Fig. 3C) (Frankenberger and Dick^48^). The relative growth rate increased linearly with urease activity in the marine microalgae species *Prorocentrum minimum* (Fig. 3D). Urease activities of *P*. *minimum* cultures grown with urea and NH4^+^ sources at the exponential growth phase were used to obtain this relationship (data from Fan *et al*.^57^).

**Figure 4:**
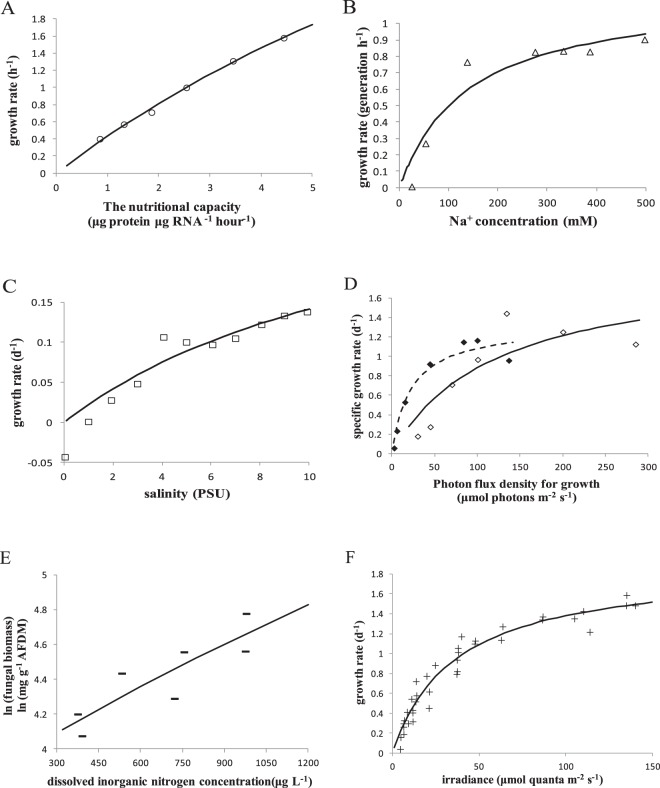
The limiting resource dependence of growth rate or natural logarithmic biomass in microbes, following Eqs (9A) and (9B). (**A**) The nutritional capacity *vs* the mass-specific growth rate of *Escherichia coli* K12 strain MG1655 (hollow circle) in medium without antibiotics (data from Scott *et al*.^58^). (**B**) Na^+^ concentration *vs* the relative growth rate of the marine bacterium *Pseudomonas doudoroffii* 70 (hollow triangle) in minimal medium, with succinate as a carbon source (data from Wisse and Macleod^59^). (**C**) Salinity *vs* the relative growth rate of a cyanobacterium, *Nodularia*. *spumigena* (hollow square) (data from Rakko and Seppälä^60^). (**D**) Photon flux *vs* the specific growth rate of *Dunaliella*. *tertiolect*a (hollow diamond) and *Phaeodactylum*. *tricornutum* (solid diamond) (data from Quigg and Beardall6^61^). (**E**) Dissolved inorganic nitrogen concentration *vs* fungal biomass associated with maple leaves (solid short line segments) (data from Burns^54^). (**F**) Irradiance *vs* growth rate for the marine diatom *Thalassiosira pseudonana* (plus sign) (data from Berges and Harrison^62^). The parameter values are given in *SI Appendix* Table S3.

The limiting resource-dependent equations of natural logarithmic biomass (Eq. 9A) and specific growth rate (Eq. 9B) were supported by data compiled from many papers. There is a curvilinear relationship between limiting resources and specific growth rate or natural logarithmic biomass (Fig. 4). The specific growth rate increased with nutritional capacity following the dynamics described by Eq. (9B) (Fig. 4A). RNA and protein extracted from the medium of strains derived from the *Escherichia coli* K12 strain MG1655 were used to calculate the nutritional capacity and mass-specific growth rate, respectively (data from Scott *et al*.^58^). The relationship between the relative exponential-state growth rate of a marine bacterium (*Pseudomonas doudoroffii* 70) and Na^+^ concentration is curvilinear (Fig. 4B). The *P*. *doudoroffii* 70 was cultured in minimal medium with succinate was added as a carbon source (data from Wisse and Macleod^59^). The specific growth rate of a Baltic Sea filamentous cyanobacterial species (*Nodularia spumigena*) in the exponential period increased only with salinity varying from 0 to 10 PSU following the dynamics described by Eq. (9B) (Fig. 4C) (data from Rakko and Seppälä^60^). The specific growth rates of two marine microalgae (*Dunaliella tertiolecta* and *Phaeodactylum tricornutum*) exhibited a curvilinear response to photon flux for growth (Fig. 4D) (data from Quigg and Beardall6^61^). Maple leaves were sampled on days 14, 28, 44, 77, and 111 (data from Burns^54^). The natural logarithmic fungal biomass associated with decaying plant litter changed with increasing dissolved inorganic nitrogen concentrations (Fig. 4E). The steady-state growth rate of a marine diatom (*Thalassiosira pseudonana*) increased with irradiance following the dynamics described by Eq. (9B) (Fig. 4F) (data from Berges and Harrison^62^).

### The natural logarithmic model with interdependence between microbial growth and metabolism

**Figure 5:**
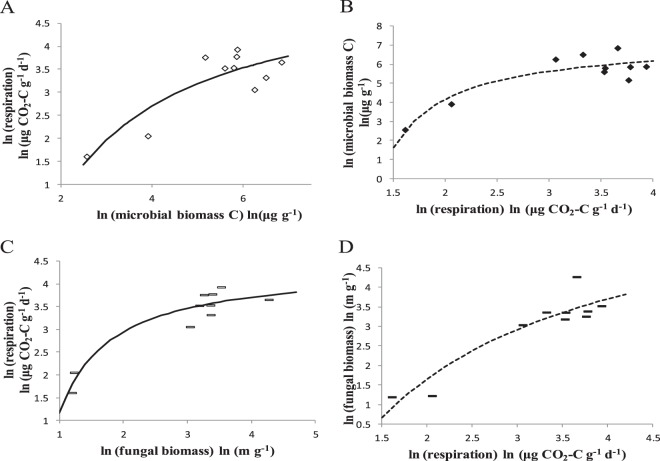
The natural logarithmic interdependent relationship between microbial biomass and metabolism, following Eq. (10A) and (10B). (**A**,**B**) In two Gray Luvisolic soil zones of Saskatchewan: the Equation (10) prediction is shown by a solid curved line (hollow diamond) and a dashed curved line (solid diamond) (data from Vvsr *et al*.^51^). (**C**,**D**) In two Grey Luvisolic soil zone of Saskatchewan: Equation (10) prediction is shown by solid curved line (hollow short line segments) and dash curved line (solid short line segments) (data from Vvsr *et al*.^51^). The parameter values that provide this best fit are given in *SI Appendix* Table S4.

The interdependent relationship between microbial metabolism and biomass or specific growth rate in natural logarithmic space (Eqs 10A and 10B) was tested by data compiled from Vvsr *et al*.^51^ (Fig. 5). In two Gray Luvisolic soil zones of Saskatchewan, microbial biomass and respiration rate exhibited a curvilinear relationship (Fig. 5A,B). At the same locations, the relationship between the fungal biomass and respiration rate also showed a curve similar to that in Fig. 5A–D).

Three statistical parameters, namely, the goodness of fit (R^2^), residual sum of squares (RSS), and Akaike’s information criterion (AIC)^63,64^, are regarded as the criterion with which to determine which model is the best representation of a curve. The ln*λ* values were regressed with respect to *M* or ln*M* using exponential, power and mass-dependent equations using the data shown in Fig. 5A,C. The power equation yielded slightly lower AIC values than mass-dependent functions we proposed here (Eq. 10A) (9.0983 < 10.9416; 1.0294 < 1.1482), but our model produced lower RSS values (1.342 < 1.665; 0.504 < 0.743) and higher R^2^ values (0.7552 > 0.6963; 0.9081 > 0.8644) (Table 1). Therefore, we argue that our model (Eq. 10A) is better than the traditional power equation (Eq. 3) based on the values of R^2^, AIC, and RSS.

**Table 1:** The comparison of model application results for microbial metabolic rate and biomass.

Model	Calculated equation	RSS		R^2^	AIC
Exponential function	$\mathrm{ln}\,\lambda =\,\mathrm{ln}\,a^{\prime} +b^{\prime} M$	Fig. 5A	122.9	0.0656	52.1137
		Fig. 5C	2.859	0.4785	14.5047
Power function	$\mathrm{ln}\,\lambda =\,\mathrm{ln}\,a^{\prime} +b^{\prime} \mathrm{ln}\,M$	Fig. 5A	1.665	0.6963**	9.0983
		Fig. 5C	0.743	0.8644***	1.0294
Mass-dependent function	$\mathrm{ln}\,\lambda =\frac{a^{\prime} \ast (\mathrm{ln}\,M-b^{\prime} )}{c^{\prime} +(\mathrm{ln}\,M-b^{\prime} )}+d^{\prime}$	Fig. 5A	1.342	0.7552***	10.9416
		Fig. 5C	0.504	0.9081***	1.1482

**Present: p < 0.01; ***present: p < 0.001.

## Discussion

Our Eqs (5), (6), (8), (9), and (10) satisfactorily characterized microbial data. The analytical results support our hypothesis and predictions (Table 1 and *SI Appendix*). The relative rates of microbial growth and metabolism are interdependent. The driver of metabolic scaling may be enzymatic dynamics rather than the ratio of surface area to volume or a fractal resource transport network^21,46,47^.

The most basic indicator of metabolism is the metabolic rate (*λ*); the metabolism and body size (body mass or biomass, M) of organisms scale as *λ* ∝ M^*α*^^20^. In the late 1990s, Rubner first described the quantitative relationship between metabolic rate and body size^38^; since then, many mathematical scaling models of metabolism have been developed to explain this allometric relationship. One of the famous models is a fractal-like distribution network (WBE) model^21,46,47^, in which the physicist West and ecologists Brown and Enquist summarized the circulatory system of animals and the vascular bundle system of plants into a resource supply network (WEB model) with self-similar structure, explaining Kleiber’s law (*α* = 3/4), in 1997. Another famous model is the metabolic-level boundaries (MLB) model proposed by Glazier which based on physical limits, explains why the exponent *α* varies from 2/3 to 1^65,66^. In addition, there are many other models to explain the different exponential values, such as efficient transportation networks^67^, cell optimization growth theory (*α* = 2/3 to 1)^68^, structural theory (large endotherm:*α* = 3/4; ectotherm:*α* = 2/3; small endotherm:*α* = 1/3 to 3/4)^69^, and energy consumption (small and medium animals:*α* = 3/4; large animals:*α* = 1)^70^. First, what these theories have in common is that the relationship between metabolism and body mass is linear in the logarithmic space. Because of the limitation of employing linear regression, these methods produce only isolated exponential values (the slopes of the lines). However, we can obtain a continuously changing dynamic for the exponent *α* because we obtained a curvilinear relationship between microbial metabolism and biomass. When we used operations of partial derivatives with respect to our curve (∂ln*λ*/∂(ln*M*-ln*c*_1_)), we obtained a continuous exponential data set containing the slopes of all the tangent lines tangent to this curve. Second, these theories mentioned above usually borrowed concepts from mathematical geometry, such as fractal geometry or physical limits^21,38,65–70^ to explain the allometric relationship and paid relatively little attention to the essence of metabolism, namely, that it is a series of enzymatic reactions. However, we argue that enzyme activities drive the relationship between microbial metabolism and biomass because metabolism and growth are a series of biochemical reactions that furnish the materials and energy necessary for biological growth, development, reproduction and evolution. In addition the data fitted by Eqs (5), (6), (8) and (9) (Figs 1–4) supported our hypothesis and predictions that enzymes drive the relative rate of both microbial metabolism and growth. We do not deny that various theories proposed above, such as the ratio of surface area to volume or the fractal resource transport network, may also affect scaling dynamics by regulating the energy balance or substrate concentration; we simply note that they do not take into account the importance and driving force of the key enzymes. Furthermore, the investigation of Miettinen and Björklund suggested that the mevalonate pathway activity which is a metabolic pathway essential for synthesizing isopentenyl pyrophosphate and dimethylallyl pyrophosphate in eukaryotes, archaea, and some bacteria^71,72^, contributes to the nonlinearity of the scaling between cell size and mitochondrial function^73^. Furthermore, fundamental aspects of enzyme activities could allow deviations from the traditional power law in principle, as we have proposed: the relationship between microbial metabolism and biomass is curvilinear and driven by their respective key enzymes.

The Monod equation, a microbial growth dynamics models, is an empirical equation and has the same form as the Michaelis-Menten equation^11^. As we validated here, the forms of the Monod equation and empirical growth law are actually part of the predictions of our hypothesis, that is, Equations (8) and (9). Unsurprisingly, accumulated evidences in this field^5,10^ also supports our hypothesis (Fig. 4). We provide a mechanical explanation for the relationship between microbial growth (biomass) and limiting resources, while the Monod equation did not.

Semi-logarithmic equations, in which the dependent variable is a natural logarithm, are primarily used in empirical economics^74^, and to describe the dynamics of some microbes, such as the isothermal semi-logarithmic survival curves of microorganisms and spores^75^. Nevertheless, our two resource-dependent semi-logarithmic equations, namely, Eqs (6), (9), differ from the research that directly introduced the Michaelis-Menten equation to describe the relationship between metabolic rate and limiting resources^34–37^. For example, the Michaelis-Menten equation is generally used to directly depict the gross photosynthetic rates relative to irradiance at the surface in aquatic systems^35^. López-Urrutia *et al*. extended the MTE to account for the relationship between individual gross photosynthesis and photosynthetically active radiation in the oceans using the Michaelis-Menten equation as well^34^. Sinsabaugh and Shah^36^ combined metabolic scaling theory^23^ and kinetic measures of extracellular enzyme activity to relate bacterial productivity to ^App^*V*max, which is a measure of enzyme abundance (catalytic capacity), and Aguiar-González *et al*.^37^ used the biochemical enzyme kinetic model (EKM) of respiratory oxygen consumption based on the substrate control of respiratory electron transfer systems. These are examples in which the Michaelis-Menten equation was used directly to consider the relations between metabolic rate (*λ*) and limiting resources. However, we predicted that there is a relationship between ln*λ* or ln*M* and limiting resources (Eqs 6 and 9) based on our hypothesis that both the relative metabolic rate (d*λ*/*λ*) and growth rate (d*M*/*M*)^10^ are constrained by their own rate-limiting enzymes. Equations (10) and (11) are not completely semi-logarithmic; their correlation is strong when the dependent variables are all in the natural logarithm. We proposed the concept of a relative metabolic rate or biomass, which is different from the studies mentioned above that directly used the metabolic rate.

**Figure 6:**
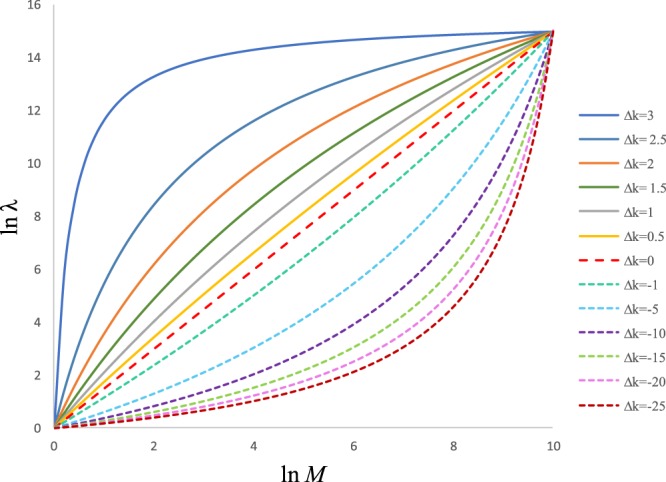
A schematic of various ∆*K* values (the difference between *K*_λ_ and *K*_*M*_) responding to different scaling curvatures.

The long-standing question of metabolic scaling may be resolved by our logarithmic equation with interdependence between metabolism and biomass. Equation (10) provides a new mechanical model for quantitatively analyzing the relationship between microbial metabolism and growth. This equation predicts that double-logarithmic dynamic shifts in the metabolism and biomass of most microbes are curvilinear, rather than linear, as predicted by the MTE and other models predicted them were under the conditions of the same types of limiting substrates in both microbial metabolism and growth and *K*_*λ*_ ≠ *K*_*M*_. Furthermore, the double-logarithmic dynamics would be linear under the conditions of the same types of limiting substrates in both microbial metabolism and growth and *K*_*λ*_ = *K*_*M*_, scaling as in Equation (11), as occurs in the power law. The smaller the difference between *K*_*λ*_ and *K*_*M*_ is, the smaller the scaling dynamic curvature is under an active state (Fig. 6). When *△K* = 0, the metabolic rate is basal, and with a shift in *△K* values, microbes need energy to sustain not only basal metabolism, but also active activities and growth. In reality, numerous datassets of the nonlinear scaling dynamics have been analyzed using the linear regression, which may be able to simplify equations, similar to the famous 3/4 power equation^20,21^. In fact, Kleiber’s law was obtained by using a strictly controlled basal metabolic rate^20^, and we presume that one of the reasons for the extensive debates about metabolic scaling is the possibility that the data used to draw conclusions, were collected imprecisely and were not completely basal. The power law is just a particular form of the natural logarithmic interdependent law rather than a general law, and our model may be more general when predicting the scaling dynamics between microbial metabolism and biomass.

## Conclusions

We hypothesized that the relative metabolic rate and growth rate might be driven by their rate-limiting enzymes in actively growing microbes. Active metabolic scaling originates from enzyme-driven processes, and the curvature of the scaling may derive from different dynamics of substrate responses between metabolism and growth. There may be a shift in the rate from the enzymatic to individual level because the relative rate of individual metabolism and growth is proportional to the rate of their respective rate-limiting-enzyme process. Thus, we conclude that natural logarithmic microbial metabolism (ln*λ*) and growth (or biomass) (ln*M*) are both dependent on limiting resources, thereby developing novel models with interdependence between ln*λ* and ln*M*, which can described the various metabolic scaling relationships in an active state with the difference between the microbial metabolic and growth (or biomass) half-saturation constants (*K*_*M*_, *K*_λ_). Moreover, under a basal state with the same values of *K*_*M*_ and *K*_λ_, there is a linear scaling relationship. The results indicate that enzymatic dynamics may be the origin of active and basal metabolic scaling, and the traditional power law is a particular case of the interdependent models under the condition *K*_*λ*_ = *K*_*M*_. Integrating the scaling law with biochemical processes helps settle various debates on the traditional power law, understand how and why the scaling relationship is usually curved, and identify what deives the degree of curvature.

## Methods

By researching for a large number of publications and by using the software GetData Graph Digitizer 2.22, we obtained relevant data for microbes, including the respiration rate, growth rate, enzyme activity, biomass, and concentration of limited resources (details provided in the Result section and *SI Appendix* Table S1), to verify our hypotheses and equations; more details are provided in the models and results. The software MATLAB R2017b was used to fit the curve and obtain all the coefficients (SI Appendix Tables S2, S3, S4).

## Supplementary information

Supporting Information and Appendix
