# Supplementary material for: Enzymatic origin and various curvatures of metabolic scaling in microbes
Source: Sci Rep. 2019 Mar 11;9:4082. doi: 10.1038/s41598-019-40712-5 (PMC6411939; doi:10.1038/s41598-019-40712-5)
Supplement: Supplementary file 1 — Supporting Information and Appendix [file 41598_2019_40712_MOESM1_ESM.pdf]

Supplementary Information and Appendix

Enzymatic origin and various curvatures of metabolic scaling in microbes

Liyan Li[1], Genxuan Wang[1]*

[1]College of Life Sciences, Zhejiang University, Hangzhou, China.

*wanggx@zju.edu.cn, 86-571-88206590.

Table S1 The information of data source used by this paper in Figures. The number of the references is the same as the text. Data items are x-axis and y-axis in respective figures, data points are the number of data in respective figures.

References	Data items obtained	Figures number	Data points
[44]Anderson *et al.*, 2017	Acid phosphatase activity<br>Soil respiration rate	Fig. 1A	11
[45]Packard *et al.*, 1983	ETS activity<br>Marine bacterial respiration rate	Fig. 1B	13
[46]Vvsr *et al.*, 1998	Dehydrogenase activity<br>Microbial respiration	Fig. 1C	10
[43]Frankenberger & Dick, 1983	Enzyme activity<br>Soil bacterial respiration	Fig. 1D	20
[47]Yuan *et al.*, 2010	Bacterial production<br>Chlorophyll a	Fig. 2A	18
[48]Manahan & Richardson, 1983	^{14}C-glycine concentration<br>Uptake rate	Fig. 2B	5
[49]Burns, 2013	Dissolved inorganic nitrogen concentration<br>Microbial respiration rates	Fig. 2C	20
[50]Imberger & Chiu, 2001	Soil ergosterol content<br>Fungal respiration	Fig. 2D	14
[51]Dar, 1996	Dehydrogenase activities<br>Soil microbial biomass	Fig. 3A	8
[45]Packard *et al.*, 1983	ETS activity<br>Marine bacterial biomass	Fig. 3B	39
[43]Frankenberger & Dick, 1983	Alkaline phosphatase activity<br>Soil microbial biomass	Fig. 3C	11
[52]Fan *et al.*, 2003	Urease activity<br>Relative growth rate	Fig. 3D	7
[53]Scott *et al.*, 2010	The nutritional capacity	Fig. 4A	6
[54]Wisse & Macleod, 1989	The mass-specific growth rate<br>Na^+ concentration<br>The relative growth rate	Fig. 4B	7
[55]Rakko & Seppälä, 2014	Salinity<br>The relative growth rate	Fig. 4C	10
[56]Quigg & Beardall, 2003	Photon flux<br>The specific growth rate	Fig. 4D	14
[49]Burns, 2013	Dissolved inorganic nitrogen concentration	Fig. 4E	7
[57]Berges & Harrison, 1993	Fungal biomass<br>Irradiance<br>Growth rate	Fig. 4F	35
[46]Vvsr et al., 1998	Microbial biomass<br>Fungal biomass<br>Microbial respiration	Fig. 5A, B<br>Fig. 5C, D	10

Table S2. The parameter values of Fig. 1A-D and Fig. 2A-D were obtained by fitting data with Eq. (5) and Eq. (6).

	a	b	ln λmax	K_λ	ln *a*	R^2
Fig.1A	7.304	0.083	-	-	-	0.7960
Fig.1B	4.170	0.112	-	-	-	0.8484
Fig.1C	18.380	0.011	-	-	-	0.5104
Fig.1D	178.100	0.011	-	-	-	0.5325
	145.700	0.010				0.5895
Fig.2A	-	-	3.150	2.403	5.066	0.5746
Fig.2B	-	-	2.712	0.010	1.593	0.9994
Fig.2C	-	-	-406.800	-1.076	413.100	0.5935
			-1205.00	-0.483	1211.000	0.4762
Fig.2D	-	-	4.041	0.157	0.157	0.9314

Table S3. The parameter values of Fig. 3A-D and Fig. 4A-F were obtained by fitting data with Eq. (8A), Eq. (8B), Eq. (9A) and Eq. (9B).

	c	h_2	h_1	μ_{max}	K_μ	ln M_{max}	K_M	ln c	R^2
Fig.3A	117.900	0.004	-	-	-	-	-	-	0.9457
Fig.3B	0.148	0.020	-	-	-	-		-	0.7073
Fig.3C	182.200	0.023	-	-	-	-	-	-	0.6847
Fig.3D	-	-	0.104	-	-	-	-	-	0.8136
Fig.4A	-	-	-	7.355	16.170	-	-	-	0.9952
Fig.4B	-	-	-	1.192	137.800	-	-	-	0.9076
Fig.4C	-	-	-	0.349	14.640	-	-	-	0.8907
Fig.4D	-	-	-	1.958	122.600	-	-	-	0.7501
				1.326	22.520				0.9495
Fig.4E	-	-	-	-	-	6.759	6703.000	3.801	0.7545
Fig.4F	-	-	-	1878.000	36.090	-	-	-	0.9471

Table S4. The parameter values of Fig. 5A-D obtained by fitting data with Eq. (10A) and Eq. (10B).

	ln λ_{maxM}	$K_{\lambda M}$	ln c_1	ln a_1	Ln$M_{max\lambda}$	$K_{M\lambda}$	ln a_2	ln c_2	R^2
Fig.5A	5.215	2.732	1.804	0.367	-	-	-	-	0.7552
Fig.5B	-	-	-	-	3.309	1.147	2.207	4.224	0.8207
Fig.5C	0.492	7.377	7.482	4.134	-	-	-	-	0.9081
Fig.5D	-	-	-	-	5.270	3.224	2.031	1.703	0.8662
